# Supplementary material for: Generation of a Dystrophin Mutant in Dog by Nuclear Transfer Using CRISPR/Cas9-Mediated Somatic Cells: A Preliminary Study
Source: Int J Mol Sci. 2022 Mar 7;23(5):2898. doi: 10.3390/ijms23052898 (PMC8911381; doi:10.3390/ijms23052898)
Supplement: Supplementary file 1 [file ijms-23-02898-s001.zip › ijms-1618897-supplementary.pdf]

International Journal of *Molecular Sciences*

MDPI

Article

Generation of a Dystrophin Mutant in Dog by Nuclear Transfer Using CRISPR/Cas9-Mediated Somatic Cells

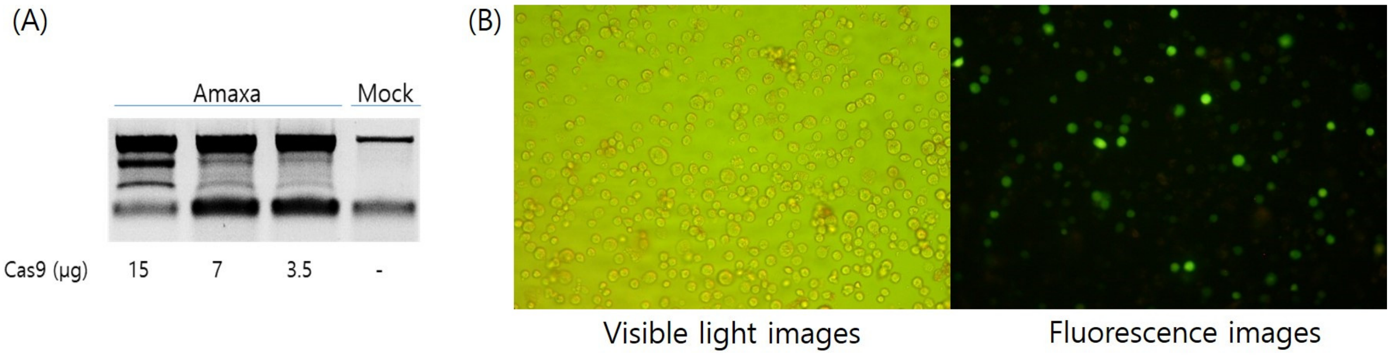

Supplemental Figure S1. Genome editing in Canine fetal fibroblast via RNP delivery. (**A**) Mutations were detected by T7E1 assay. A mixture of Cas9 protein and in vitro transcribed sgRNA (20 μg) was transfected into 2x105 cells. (**B**) Fibroblasts expressing green fluorescence protein transiently(original magnification 100X).

Total Sequences	With both indicator sequences	More than minimum frequency	Insertions	Deletions	Indel frequency	HDR frequency
71232	51673	51396	0	51396	51396 (100.0%)	0 (0.0%)

<table>
<tr><th>ID</th><th>Sequence</th><th>Length</th><th>Count</th><th>Type</th><th>HDR</th></tr>
<tr><td>1</td><td>TGAGCTGGGTCCGACAATCAACTCGTAATTATCCACAGGTTAATGTCATTAACTTCACCACCAGCTGGTCTGATGGCCTGGCTTT
TGAGCTGGGTCCGAC---ATGGCCTGGCTTT

GAACGCTCTCATCCACAGTCATAGGTAAGGAGACCACTGAGATATTGACTAATTT
GAACGCTCTCATCCACAGTCATAGGTAAGGAGACCACTGAGATATTGACTAATTT</td><td>83</td><td>47491</td><td>Del</td><td>N/A</td></tr>
<tr><td>2</td><td>TGAGCTGGGTCCGACAATCAACTCGTAATTATCCACAGGTTAATGTCATTAACTTCACCACCAGCTGGTCTGATGGCCTGGCTTT
TGAGCTGGGTCCGAC---ATGGCCTGGCTTT

GAACGCTCTCATCCACAGTCATAGGTAAGGAGACCACTGAGATATTGACTAATTT
GAACGCTCTCATCCACAGTCATAGGCAAGGAGACCACTGAGATATTGACTAATTT</td><td>83</td><td>120</td><td>Del</td><td>N/A</td></tr>
<tr><td>3</td><td>TGAGCTGGGTCCGACAATCAACTCGTAATTATCCACAGGTTAATGTCATTAACTTCACCACCAGCTGGTCTGATGGCCTGGCTTT
TGAGCTGGGTCCGAC---ATGGCCTGGCTTT

GAACGCTCTCATCCACAGTCATAGGTAAGGAGACCACTGAGATATTGACTAATTT
GAACACTCTCATCCACAGTCATAGGTAAGGAGACCACTGAGATATTGACTAATTT</td><td>83</td><td>100</td><td>Del</td><td>N/A</td></tr>
</table>

Supplemental Figure S2. Targeted mutagenesis in dog cells by CRISPR/Cas9. The result of deep sequencing of mutant dog cells. Target Sequence of the sgRNA is underlined within the genomic sequence of DMD gene. Red boxes represent a protospacer-adjacent motif (PAM) sequence.

Mismatch 0 ; 1 / Mismatch 1 ; 0 / Mismatch 2 ; 2 / Mismatch 3 ; 16

	Target	Chromosome	Position	Direction	Mismatches	
1	crRNA: TTCACCACCAGCTGGTCTGANGG<br>DNA: cTCtCCACCAGCTGGTCTGgGGG	chr24	22878368	+	3	
2	crRNA: TTCACCACCAGCTGGTCTGANGG<br>DNA: TTCACCtCCAGCTGtTCTaATGG	chr8	29061641	+	3	
3	crRNA: TTCACCACCAGCTGGTCTGANGG<br>DNA: TTCACaAgCAGCTGGaCTGAGGG	chr12	4938755	+	3	
4	crRNA: TTCACCACCAGCTGGTCTGANGG<br>DNA: TTCACCACattCTGGTCTGAAGG	chr3	26238745	+	3	
5	crRNA: TTCACCACCAGCTGGTCTGANGG<br>DNA: TTCACCACCAcCTGGcCTGATGG	chr3	50964839	-	2	
6	crRNA: TTCACCACCAGCTGGTCTGANGG<br>DNA: cgCcCCACCAGCTGGTCTGACGG	chr3	57426757	+	3	
7	crRNA: TTCACCACCAGCTGGTCTGANGG<br>DNA: TTCACCcCCAGCTGaTCTGgGGG	chr3	62735685	+	3	
8	crRNA: TTCACCACCAGCTGGTCTGANGG<br>DNA: TTCACCAgCAGCTGGTaTGgTGG	chr3	73060142	+	3	
9	crRNA: TTCACCACCAGCTGGTCTGANGG<br>DNA: aTCAgCACCAGCTGGgCTGAGGG	chr25	45357425	-	3	
10	crRNA: TTCACCACCAGCTGGTCTGANGG<br>DNA: TTCACCACCAcacGGTCTGAAGG	chr1	16905540	-	3	
11	crRNA: TTCACCACCAGCTGGTCTGANGG<br>DNA: TTCACCACCAGCTGGaCaGATGG	chr1	35850847	+	2	
12	crRNA: TTCACCACCAGCTGGTCTGANGG<br>DNA: TTCACCACCAGCTGGaggGATGG	chr1	1.13E+08	-	3	
13	crRNA: TTCACCACCAGCTGGTCTGANGG<br>DNA: TTCAgCAtCAGCTGGcCTGACGG	chr33	17707648	+	3	
14	crRNA: TTCACCACCAGCTGGTCTGANGG<br>DNA: TTCACCAtCAGCgGGTCTGtAGG	chr20	56928844	+	3	
	crRNA: TTCACCACCAGCTGGTCTGANGG<br>DNA: TTCACCACCAGCTGGTCTGATGG	chrX	27933928	-	0	On target
15	crRNA: TTCACCACCAGCTGGTCTGANGG<br>DNA: aTCACCACCAGCTGagCTGAAGG	chrX	1.06E+08	+	3	
16	crRNA: TTCACCACCAGCTGGTCTGANGG<br>DNA: aTCACCACCAGCTGagCTGAAGG	chrX	1.06E+08	-	3	
17	crRNA: TTCACCACCAGCTGGTCTGANGG<br>DNA: TTCACCACCAGCTGGagaGATGG	chr18	50645543	+	3	
18	crRNA: TTCACCACCAGCTGGTCTGANGG<br>DNA: TgCACCACCAGCaGGTCTGcAGG	chr28	32426415	-	3	

Supplemental Figure S3. Potential off-target sites with up to 3 mismatches, relative to the wild-type sequence.

Off-target Analysis

	Total Sequences	With both indicator sequences	More than minimum frequency	Insertions	Deletions	Indel frequency
**1**	13213	11325	10553	0	68	68 (0.6%)
**2**	14376	13779	13442	0	7	7 (0.1%)
**3**	12183	11326	10835	0	9	9 (0.1%)
**4**	15741	15231	14892	0	18	18 (0.1%)
**5**	15043	14459	14088	0	2	2 (0.0%)
**6**	14224	12056	11843	0	4	4 (0.0%)
**7**	11372	10141	9789	0	3	3 (0.0%)
**8**	12832	12439	12244	0	2	2 (0.0%)
**9**	330652	11629	11439	0	2	2 (0.0%)
**10**	339871	8548	8240	0	2	2 (0.0%)
**11**	336445	9669	9364	0	0	0 (0.0%)
**12**	295067	8274	8135	0	0	0 (0.0%)
**13**	245902	9766	9577	0	0	0 (0.0%)
**14**	280667	9419	8966	0	0	0 (0.0%)
**15**	256539	10355	10007	0	0	0 (0.0%)
**16**	235573	9310	8534	0	0	0 (0.0%)
**17**	337899	8469	8107	0	0	0 (0.0%)
**18**	373015	7996	7691	0	0	0 (0.0%)

#6

```
1  GTTTGTTTAGCCGCCCCACCAGCTGGTCTGACGGCCAGGTCCTATGCTCGCTCTTGCTGGCCATCCATTCCCCCAGCA   78   6126   WT or Sub
   ||||||||||||.|||||||||||||||||||||||||||||||||||||||||||||||||||||||||||||||||
   GTTTGTTTAGCCTCCCCACCAGCTGGTCTGACGGCCAGGTCCTATGCTCGCTCTTGCTGGCCATCCATTCCCCCAGCA

2  GTTTGTTTAGCCGCCCCACCAGCTGGTCTGACGGCCAGGTCCTATGCTCGCTCTTGCTGGCCATCCATTCCCCCAGCA   78   4872   WT or Sub
   ||||||||||||||||||||||||||||||||||||||||||||||||||||||||||||||||||||||||||||||
   GTTTGTTTAGCCGCCCCACCAGCTGGTCTGACGGCCAGGTCCTATGCTCGCTCTTGCTGGCCATCCATTCCCCCAGCA
```

Supplemental Figure S4. No off-target mutations at candidate sites in dystrophin mutant dog. Indel frequencies at potential off-target sites were analyzed using targeted deep sequencing. Off-target candidate #6 had 1 bp substituted allele which is pre-existed SNP not caused by CRISPR/Cas9.

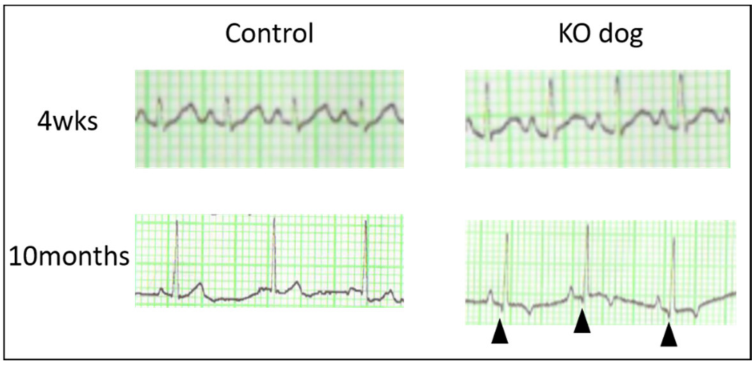

Supplemental Figure S5. Electrocardiogram changes according to age in normal and *dystrophin* mutant dogs. Arrows indicate the Q wave in the dystrophin mutant dog.

(A)

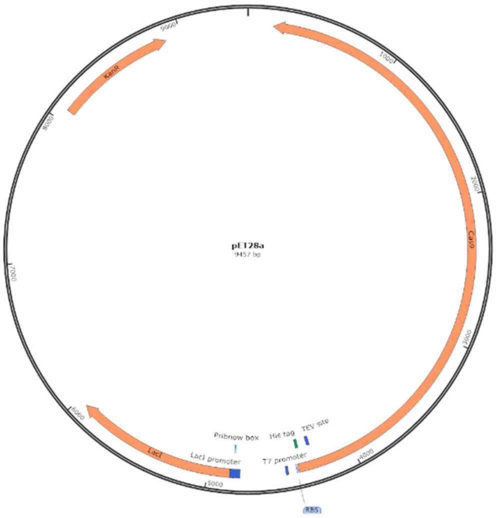

(B)

agtgccatgtccggttttcaacaaaccatgcaaatgctgaatgagggcatcgttcccactgcgatgctggttgccaacga
tcagatggcgctgggcgcaatgcgcgccattaccgagtccgggctgcgcgttggtgcggatatctcggtagtgggatacg
acgataccgaagacagctcatgttatatcccgccgttaaccaccatcaaacaggattttcgcctgctggggcaaaccagc
gtggaccgcttgctgcaactctctcagggccaggcggtgaagggcaatcagctgttgcccgtctcactggtgaaaagaaa
aaccaccctggcgcccaatacgcaaaccgcctctccccgcgcgttggccgattcattaatgcagctggcacgacaggttt
cccgactggaaagcgggcagtgagcgcaacgcaattaatgtaagttagctcactcattaggcaccgggatctcgaccgat
gcccttgagagccttcaacccagtcagctccttccggtgggcgcggggcatgactatcgtcgccgcacttatgactgtct
tctttatcatgcaactcgtaggacaggtgccggcagcgctctgggtcattttcggcgaggaccgctttcgctggagcgcg
acgatgatcggcctgtcgcttgcggtattcggaatcttgcacgccctcgctcaagccttcgtcactggtcccgccaccaa
acgtttcggcgagaagcaggccattatcgccggcatggcggccccacgggtgcgcatgatcgtgctcctgtcgttgagga
cccggctaggctggcggggttgccttactggttagcagaatgaatcaccgatacgcgagcgaacgtgaagcgactgctgc
tgcaaaacgtctgcgacctgagcaacaacatgaatggtcttcggtttccgtgtttcgtaaagtctggaaacgcggaagtc
agcgccctgcaccattatgttccggatctgcatcgcaggatgctgctggctaccctgtggaacacctacatctgtattaa
cgaagcgctggcattgaccctgagtgatttttctctggtcccgccgcatccataccgccagttgtttaccctcacaacgt
tccagtaaccgggcatgttcatcatcagtaacccgtatcgtgagcatcctctctcgtttcatcggtatcattacccccat
gaacagaaatcccccttacacggaggcatcagtgaccaaacaggaaaaaaccgcccttaacatggcccgctttatcagaa
gccagacattaacgcttctggagaaactcaacgagctggacgcggatgaacaggcagacatctgtgaatcgcttcacgac
cacgctgatgagctttaccgcagctgcctcgcgcgtttcggtgatgacggtgaaaacctctgacacatgcagctcccgga
gacggtcacagcttgtctgtaagcggatgccgggagcagacaagcccgtcagggcgcgtcagcgggtgttggcgggtgtc
ggggcgcagccatgacccagtcacgtagcgatagcggagtgtatactggcttaactatgcggcatcagagcagattgtac
tgagagtgcaccatatatgcggtgtgaaataccgcacagatgcgtaaggagaaaataccgcatcaggcgctcttccgctt
cctcgctcactgactcgctgcgctcggtcgttcggctgcggcgagcggtatcagctcactcaaaggcggtaatacggtta
tccacagaatcaggggataacgcaggaaagaacatgtgagcaaaaggccagcaaaaggccaggaaccgtaaaaaggccgc
gttgctggcgtttttccataggctccgcccccctgacgagcatcacaaaaatcgacgctcaagtcagaggtggcgaaacc
cgacaggactataaagataccaggcgtttccccctggaagctccctcgtgcgctctcctgttccgaccctgccgcttacc
ggatacctgtccgcctttctcccttcgggaagcgtggcgctttctcatagctcacgctgtaggtatctcagttcggtgta
ggtcgttcgctccaagctgggctgtgtgcacgaaccccccgttcagcccgaccgctgcgccttatccggtaactatcgtc
ttgagtccaacccggtaagacacgacttatcgccactggcagcagccactggtaacaggattagcagagcgaggtatgta
ggcggtgctacagagttcttgaagtggtggcctaactacggctacactagaaggacagtatttggtatctgcgctctgct
gaagccagttaccttcggaaaaagagttggtagctcttgatccggcaaacaaaccaccgctggtagcggtggtttttttg
tttgcaagcagcagattacgcgcagaaaaaaaggatctcaagaagatcctttgatcttttctacggggtctgacgctcag
tggaacgaaaactcacgttaagggattttggtcatgaacaataaaactgtctgcttacataaacagtaatacaaggggtg
ttatgagccatattcaacgggaaacgtcttgctctaggccgcgattaaattccaacatggatgctgatttatatgggtat
aaatgggctcgcgataatgtcgggcaatcaggtgcgacaatctatcgattgtatgggaagcccgatgcgccagagttgtt
tctgaaacatggcaaaggtagcgttgccaatgatgttacagatgagatggtcagactaaactggctgacggaatttatgc
ctcttccgaccatcaagcattttatccgtactcctgatgatgcatggttactcaccactgcgatccccgggaaaacagca
ttccaggtattagaagaatatcctgattcaggtgaaaatattgttgatgcgctggcagtgttcctgcgccggttgcattc
gattcctgtttgtaattgtccttttaacagcgatcgcgtatttcgtctcgctcaggcgcaatcacgaatgaataacggtt
tggttgatgcgagtgattttgatgacgagcgtaatggctggcctgttgaacaagtctggaaagaaatgcataaacttttg
ccattctcaccggattcagtcgtcactcatggtgatttctcacttgataaccttatttttgacgaggggaaattaatagg
ttgtattgatgttggacgagtcggaatcgcagaccgataccaggatcttgccatcctatggaactgcctcggtgagtttt
ctccttcattacagaaacggctttttcaaaaatatggtattgataatcctgatatgaataaattgcagtttcatttgatg
ctcgatgagtttttctaagaattaattcatgagcggatacatatttgaatgtatttagaaaaataaacaaataggggttc
cgcgcacatttccccgaaaagtgccacctgaaattgtaaacgttaatattttgttaaaattcgcgttaaatttttgttaa
atcagctcattttttaaccaataggccgaaatcggcaaaatcccttataaatcaaaagaatagaccgagatagggttgag
tgttgttccagtttggaacaagagtccactattaaagaacgtggactccaacgtcaaagggcgaaaaaccgtctatcagg
gcgatggcccactacgtgaaccatcaccctaatcaagttttttggggtcgaggtgccgtaaagcactaaatcggaaccct
aaagggagcccccgatttagagcttgacggggaaagccggcgaacgtggcgagaaaggaagggaagaaagcgaaaggagc
gggcgctagggcgctggcaagtgtagcggtcacgctgcgcgtaaccaccacacccgccgcgcttaatgcgccgctacagg
gcgcgtcccattcgcca

Supplemental Figure S6. pET vector. (**A**) vector map (**B**) vector sequence

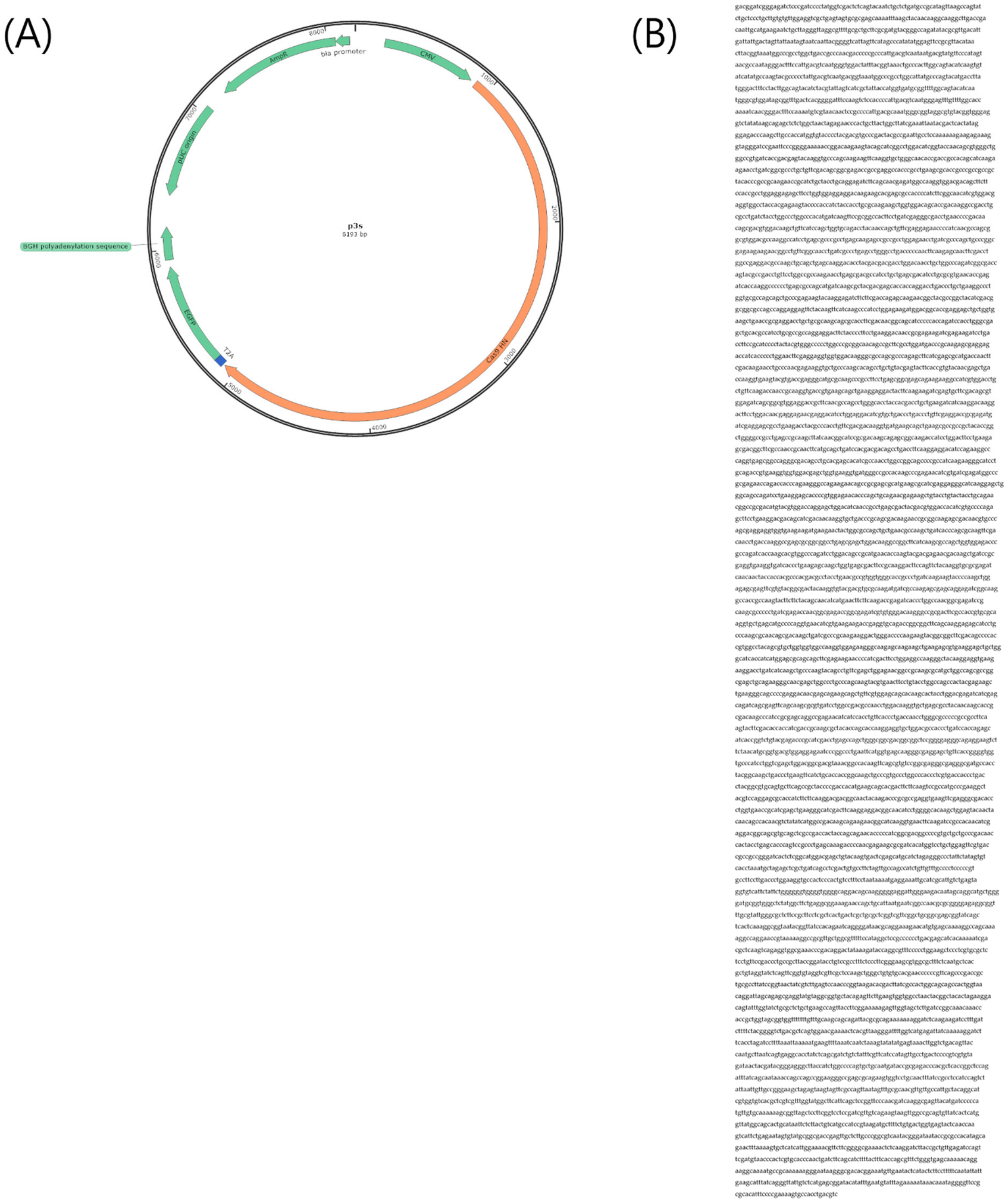

Supplemental Figure S7. p3s-Cas9-2A-GFP vector vector. (**A**) vector map (**B**) vector sequence

(A)

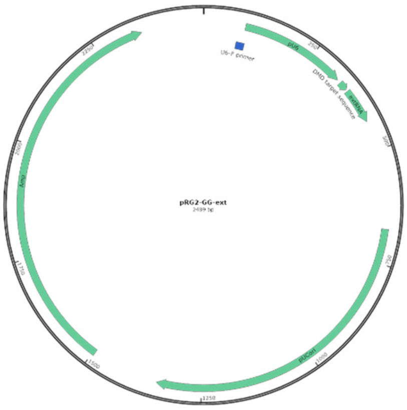

(B)

gacgaagactcaattgtcgattagtgaacggatctcgacggtatcgatcacgagactagcctcgagcggccgccccctt c
accgagggcctatttcccatgattccttcatatttgcatatacgatacaaggctgttagagagataattggaattaattt
gactgtaaacacaaagatattagtacaaaatacgtgacgtagaaagtaataatttcttgggtagtttgcagttttaaaat
tatgttttaaaatggactatcatatgcttaccgtaacttgaaagtatttcgatttcttggctttatatatcttgtggaaa
ggacgaaacaccgttcaccaccagctggtctgagttttagagctagaaatagcaagttaaaataaggctagtccgttatc
aacttgaaaaagtggcaccgagtcggtgctttttttctagattcgcgatgtacgggccagatatacgcgttgacattgatt
attgactagttgtcttcctgcattaatgaatcggccaacgcgcggggagaggcggtttgcgtattgggcgctcttccgct
tcctcgctcactgactcgctgcgctcggtcgttcggctgcggcgagcggtatcagctcactcaaaggcggtaatacggtt
atccacagaatcaggggataacgcaggaaagaacatgtgagcaaaaggccagcaaaaggccaggaaccgtaaaaaggccg
cgttgctggcgtttttccataggctccgcccccctgacgagcatcacaaaaatcgacgctcaagtcagaggtggcgaaac
ccgacaggactataaagataccaggcgtttccccctggaagctccctcgtgcgctctcctgttccgaccctgccgcttac
cggatacctgtccgcctttctcccttcgggaagcgtggcgctttctcaatgctcacgctgtaggtatctcagttcggtgt
aggtcgttcgctccaagctgggctgtgtgcacgaaccccccgttcagcccgaccgctgcgccttatccggtaactatcgt
cttgagtccaacccggtaagacacgacttatcgccactggcagcagccactggtaacaggattagcagagcgaggtatgt
aggcggtgctacagagttcttgaagtggtggcctaactacggctacactagaaggacagtatttggtatctgcgctctgc
tgaagccagttaccttcggaaaaagagttggtagctcttgatccggcaaacaaaccaccgctggtagcggtggtttttt
gtttgcaagcagcagattacgcgcagaaaaaaaggatctcaagaagatcctttgatcttttctacggggtctgacgctca
gtggaacgaaaactcacgttaagggattttggtcatgagattatcaaaaaggatcttcacctagatccttttaaattaaa
aatgaagttttaaatcaatctaaagtatatatgagtaaacttggtctgacagttaccaatgcttaatcagtgaggcacct
atctcagcgatctgtctatttcgttcatccatagttgcctgactccccgtcgtgtagataactacgatacgggagggctt
accatctggccccagtgctgcaatgataccgcgagatccacgctcaccggctccagatttatcagcaataaaccagccag
ccggaagggccgagcgcagaagtggtcctgcaactttatccgcctccatccagtctattaattgttgccgggaagctaga
gtaagtagttcgccagttaatagtttgcgcaacgttgttgccattgctacaggcatcgtggtgtcacgctcgtcgtttgg
tatggcttcattcagctccggttcccaacgatcaaggcgagttacatgatcccccatgttgtgcaaaaaagcggttagct
ccttcggtcctccgatcgttgtcagaagtaagttggccgcagtgttatcactcatggttatggcagcactgcataattct
cttactgtcatgccatccgtaagatgcttttctgtgactggtgagtactcaaccaagtcattctgagaatagtgtatgcg
gcgaccgagttgctcttgcccggcgtcaatacgggataataccgcgccacatagcagaactttaaaagtgctcatcattg
gaaaacgttcttcggggcgaaaactctcaaggatcttaccgctgttgagatccagttcgatgtaacccactcgtgcaccc
aactgatcttcagcatcttttactttcaccagcgtttctgggtgagcaaaaacaggaaggcaaaatgccgcaaaaaaggg
aataagggcgacacggaaatgttgaatactcatactcttcctttttcaatattattgaagcatttatcagggttattgtc
tcatgagcggatacatatttgaatgtatttagaaaaataaacaaataggggttccgcgcacatttccccgaaaagtgcca
cctgacgtc

Supplemental Figure S8. pRG2-sgRNA vector. (**A**) vector map (**B**) vector sequence

Supplemental Table S1. Parameters of MRI sequences.

	T1 Axial	T1 Axial CE	T2 Coronal	T2 Axial	T2 IDEAL
Sequence	FSE	FSE	FSE	FSE	FSE
FOV (cm)	18 × 18	18 × 18	18 × 18	18 × 18	18 × 18
Slice thickness(mm)	3	3	3	3	3
Interslice gap (mm)	0.3	0.3	0.3	0.3	0.3
Relaxation time (ms)	759	1478	4315	5313	7313
Echo time (ms)	10.1	10.1	71.6	71.8	66.6
Matrix	256 × 192	256 × 192	256 × 192	256 × 192	256 × 224
Nex	4.00	4.00	4.00	4.00	3.00

CE, contrast enhanced; FSE, fast spin echo; FOV, field of view

Supplemental Table S2. T2 values of each hind-limb muscle

T2 values	Control dog	*Dystrophin* mutant dog
Rectus femoris	38.1	62.8
Vastus lateralis	36.7	41.9
Vastus medialis	41.5	45.4
Biceps femoris	40	39.5
Semitendinosus	37.3	41.4
Semimembranosus	37.1	40.6
Adductor magnus	40.3	49.3
Mean ± SD	38.7 ± 1.9	45.8 ± 8.2

SD, standard deviation

Supplemental Table S3. List of primers used for targeted deep sequencing

**Primers used for T7E1 assay and Deep Seqeuncing of on-target site**	
Fwd_1st	GACACCTACCAATCAGAGTAGATTCC
Rev_1st	CAGTGGATAGTCAGATCAGTATGG
Fwd_2nd	GAATGGACTCCGTCCTGGTAG
Rev_2nd	GAAATGAGCTGGAACCACACTGG
Fwd_DS	ACACTCTTTCCCTACACGACGCTCTTCCGATCTTGGATTGCAACAAACCAACA
Rev_DS	GTGACTGGAGTTCAGACGTGTGCTCTTCCGATCTTGGAACCACACTGGTGAAAA
**Primers used for Deep Sequencing of off-target site**	
DogDMD_Off_1_O_F	AGGACACCCAGGTCCTTACC
DogDMD_Off_1_O_R	CCACTGTGTTCTTCCCAGGT
DogDMD_Off_2_O_F	TGCACCTGGTCTTAGCTCCT
DogDMD_Off_2_O_R	TCCTCCTTCTGCCTGTGTCT
DogDMD_Off_3_O_F	GACTCAATCCCAGGTCTCCA
DogDMD_Off_3_O_R	CCATTTCGGCAGAAAATCAT
DogDMD_Off_4_O_F	GCTGGATCCTGGGTAGACAA
DogDMD_Off_4_O_R	CCAATGTGGGACTTGATCCT
DogDMD_Off_5_O_F	TCACCTAGGCATGCATTCAA
DogDMD_Off_5_O_R	CCCCCTGGTTGACTAATGTG
DogDMD_Off_6_O_F	CAGCTCTGAAGGACCACACA
DogDMD_Off_6_O_R	CAGCAGGTAAACACCAAGCA
DogDMD_Off_7_O_F	CATGGAGGACACGATGCTAA
DogDMD_Off_7_O_R	GTGCAGGCAGCAACTTGTAA
DogDMD_Off_8_O_F	AAACTCCTTTTTGGGGCCTA
DogDMD_Off_8_O_R	TCCTCCTCTCATCTGGGCTA
DogDMD_Off_9_O_F	GGGATTTTGTTCCCAAGGTT
DogDMD_Off_9_O_R	GAGAAGAATTTGGGGGAAGC
DogDMD_Off_10_O_F	AACCGCAAACCAATACAAGG
DogDMD_Off_10_O_R	GGGAAAGACAGACCTTCACG
DogDMD_Off_11_O_F	TAGGGTTGCCATCACAAGGT
DogDMD_Off_11_O_R	TTCAGGAATGTCCTGGCTCT
DogDMD_Off_12_O_F	AGAGTACATGGCCTGGATGG
DogDMD_Off_12_O_R	GATGGAGACTGGGCTCTCTG
DogDMD_Off_13_O_F	TCCCATGAAAGAGGATCCAG
DogDMD_Off_13_O_R	TGTGTGGTCTCCCCTGTGTA
DogDMD_Off_14_O_F	TTGCAGTGGTGGAGAGAGTG
DogDMD_Off_14_O_R	TGTGGGTCACAGAACTGGAA
DogDMD_Off_15_O_F	TTGGTTTGGTTGGTGCAGTA
DogDMD_Off_15_O_R	CAGTTGACGGCAGTTGAGAA
DogDMD_Off_16_O_F	CAGTTGATGGCAGTTGAGAA
DogDMD_Off_16_O_R	TTGGTTTGGTTGGTGCAGTA
DogDMD_Off_17_O_F	CCATCCATCATCCTTTGCTT
DogDMD_Off_17_O_R	TGAAAGCGTTTTGCAGATTG
DogDMD_Off_18_O_F	GCTTCTTGGAGGAGCAACAC
DogDMD_Off_18_O_R	CAGGGAACAGGCTGTGGTAT
DogDMD_Off_1_i_F	CCAGGACAGCAGGAAGAAAG
DogDMD_Off_1_i_R	GCCTCCTGGTGGATTATGAA
DogDMD_Off_2_i_F	CCTCTTGATGAGTCCCATTGA
DogDMD_Off_2_i_R	GGGAAACTTTTGTTTGCCTTT

DogDMD_Off_3_i_F	CTCACTTCAGGCTTGCCTCT
DogDMD_Off_3_i_R	TCCCCTCACCTTGACTTCAG
DogDMD_Off_4_i_F	TGCCTTAACCATTTCTATCCTCA
DogDMD_Off_4_i_R	GCGTCCCCCTGTTTAATCTT
DogDMD_Off_5_i_F	TTCTACCCAAAGCAGCCACT
DogDMD_Off_5_i_R	TCCAAACCTTTCCTTGTGCT
DogDMD_Off_6_i_F	CCTGTGGTCTGGTGACATTG
DogDMD_Off_6_i_R	CACACCTGACACTTGGCTGT
DogDMD_Off_7_i_F	CAAGCCAGACACAAAAGCAA
DogDMD_Off_7_i_R	CACCCAATGCCTAGCTCTTC
DogDMD_Off_8_i_F	AGGAAAAGTTGGGACGGTCT
DogDMD_Off_8_i_R	CAGGTTCCTACCTCGCTCTG
DogDMD_Off_9_i_F	TTCACAACCCCGTGGATAAT
DogDMD_Off_9_i_R	TGTGTGGATCAGAGCAGAGG
DogDMD_Off_10_i_F	CCCCAGTCACTGATCCCTTA
DogDMD_Off_10_i_R	GAGCTCACGGCTGGACTTAC
DogDMD_Off_11_i_F	CTTTTGAGGCTTCCCTCCTT
DogDMD_Off_11_i_R	GACACTGCACTGTGGCATTT
DogDMD_Off_12_i_F	GGGTCCTCGCATCTATGTGT
DogDMD_Off_12_i_R	CCCTTCTTTCTTTCCCCATC
DogDMD_Off_13_i_F	CATCCCTGAACACTCCTGGT
DogDMD_Off_13_i_R	CAGGTTTGGAGGATCTTGGA
DogDMD_Off_14_i_F	CTGGGAGACACTGTGCGTAG
DogDMD_Off_14_i_R	GAGGGAAACGGACAGCACTA
DogDMD_Off_15_i_F	CCTGTCAAAGGTGGAAGCAT
DogDMD_Off_15_i_R	TCACGCCAGTCATTTCTGAG
DogDMD_Off_16_i_F	TCACACCAGTCATTTCTGAG
DogDMD_Off_16_i_R	CCTGTCAAAGGTGGAAGCAT
DogDMD_Off_17_i_F	CTGTCCCTGCACCCCTATTA
DogDMD_Off_17_i_R	CCCAGCTCTTGTCTGGATGT
DogDMD_Off_18_i_F	CCCTCGTCCTCCAACAAATA
DogDMD_Off_18_i_R	GGGGTCACACACCAGGTACT
DogDMD_Off_1_DS_F	ACACTCTTTCCCTACACGACGCTCTTCCGATCTGACCAGAATCCAAGCCTCCT
DogDMD_Off_1_DS_R	GTGACTGGAGTTCAGACGTGTGCTCTTCCGATCTCTGGACTGGTCAACTCAGCA
DogDMD_Off_2_DS_F	ACACTCTTTCCCTACACGACGCTCTTCCGATCTCAAAGACCTGGGATTCCAAA
DogDMD_Off_2_DS_R	GTGACTGGAGTTCAGACGTGTGCTCTTCCGATCTTTTCATTTAACTCAAACACAACG
DogDMD_Off_3_DS_F	ACACTCTTTCCCTACACGACGCTCTTCCGATCTGGTGTCCCTGTCTCAAGGTT
DogDMD_Off_3_DS_R	GTGACTGGAGTTCAGACGTGTGCTCTTCCGATCTGATGGGTTACAAAGGGCTGT
DogDMD_Off_4_DS_F	ACACTCTTTCCCTACACGACGCTCTTCCGATCTGGAAAAGAGAGAAGGAGGGAAA
DogDMD_Off_4_DS_R	GTGACTGGAGTTCAGACGTGTGCTCTTCCGATCTGATTTGGCTCTTTGGCTGAA
DogDMD_Off_5_DS_F	ACACTCTTTCCCTACACGACGCTCTTCCGATCTGCATTTTTCCTAAATGCCAGA
DogDMD_Off_5_DS_R	GTGACTGGAGTTCAGACGTGTGCTCTTCCGATCTTCCATTGGGGTAAAGACTGG
DogDMD_Off_6_DS_F	ACACTCTTTCCCTACACGACGCTCTTCCGATCTCTGGCCAAGGTTCTGTTTGT
DogDMD_Off_6_DS_R	GTGACTGGAGTTCAGACGTGTGCTCTTCCGATCTTGTGTGTCTGGGCTGAGTTC
DogDMD_Off_7_DS_F	ACACTCTTTCCCTACACGACGCTCTTCCGATCTGGGATGGAGTGCCAGTTTTA
DogDMD_Off_7_DS_R	GTGACTGGAGTTCAGACGTGTGCTCTTCCGATCTCAGGGAGTCCAACACAGGAC
DogDMD_Off_8_DS_F	ACACTCTTTCCCTACACGACGCTCTTCCGATCTTCACAGCAAACTACCCCAAA
DogDMD_Off_8_DS_R	GTGACTGGAGTTCAGACGTGTGCTCTTCCGATCTAGGCTTGCCAGTGAAACATC
DogDMD_Off_9_DS_F	ACACTCTTTCCCTACACGACGCTCTTCCGATCTCCTCTTGCTCCAAAGCTGTC

DogDMD_Off_9_DS_R	GTGACTGGAGTTCAGACGTGTGCTCTTCCGATCTGCTGGTAGGCTGCTCATCTC
DogDMD_Off_10_DS_F	ACACTCTTTCCCTACACGACGCTCTTCCGATCTCAAAGCCAGAAGATGGAAGC
DogDMD_Off_10_DS_R	GTGACTGGAGTTCAGACGTGTGCTCTTCCGATCTGGTGACCACCCTGGTTTCTA
DogDMD_Off_11_DS_F	ACACTCTTTCCCTACACGACGCTCTTCCGATCTGTCAGACCTGCAGCAGACAA
DogDMD_Off_11_DS_R	GTGACTGGAGTTCAGACGTGTGCTCTTCCGATCTGGGGGCTTTAGTCAGGGTAG
DogDMD_Off_12_DS_F	ACACTCTTTCCCTACACGACGCTCTTCCGATCTAGGTCAGTGGGTACCTGTGC
DogDMD_Off_12_DS_R	GTGACTGGAGTTCAGACGTGTGCTCTTCCGATCTATCTATCCACCCTCCCATCC
DogDMD_Off_13_DS_F	ACACTCTTTCCCTACACGACGCTCTTCCGATCTACCCTGGCTTGTACCCATTT
DogDMD_Off_13_DS_R	GTGACTGGAGTTCAGACGTGTGCTCTTCCGATCTAGTGCAAGCTGGAGTGTCCT
DogDMD_Off_14_DS_F	ACACTCTTTCCCTACACGACGCTCTTCCGATCTGGTTTACCTGCGTCCTCTCA
DogDMD_Off_14_DS_R	GTGACTGGAGTTCAGACGTGTGCTCTTCCGATCTCCCCCAGAAACACAGCTAAG
DogDMD_Off_15_DS_F	ACACTCTTTCCCTACACGACGCTCTTCCGATCTGAAAAGCAGGCTCCCTGTAA
DogDMD_Off_15_DS_R	GTGACTGGAGTTCAGACGTGTGCTCTTCCGATCTCATGTGAAAGTGGAGGTCCATA
DogDMD_Off_16_DS_F	ACACTCTTTCCCTACACGACGCTCTTCCGATCTCATGTGAAAGTGGAGGTCCATA
DogDMD_Off_16_DS_R	GTGACTGGAGTTCAGACGTGTGCTCTTCCGATCTGAAAAGCAGGCTCCCTGTAA
DogDMD_Off_17_DS_F	ACACTCTTTCCCTACACGACGCTCTTCCGATCTGGCTGGGCCCTACTCACTAT
DogDMD_Off_17_DS_R	GTGACTGGAGTTCAGACGTGTGCTCTTCCGATCTGTCCATCTCATGGCTCGTCT
DogDMD_Off_18_DS_F	ACACTCTTTCCCTACACGACGCTCTTCCGATCTGAGCCACTTGGTCTCCTCTG
DogDMD_Off_18_DS_R	GTGACTGGAGTTCAGACGTGTGCTCTTCCGATCTTCTCCATGCAGTCCTCACAC
